# Mediators of Inflammation – A Potential Source of Biomarkers in Oral Squamous Cell Carcinoma

**DOI:** 10.1155/2018/1061780

**Published:** 2018-11-12

**Authors:** Mircea Tampa, Madalina Irina Mitran, Cristina Iulia Mitran, Maria Isabela Sarbu, Clara Matei, Ilinca Nicolae, Ana Caruntu, Sandra Milena Tocut, Mircea Ioan Popa, Constantin Caruntu, Simona Roxana Georgescu

**Affiliations:** ^1^“Victor Babes” Clinical Hospital for Infectious Diseases, 281 Mihai Bravu, 030303 Bucharest, Romania; ^2^“Carol Davila” University of Medicine and Pharmacy, 37 Dionisie Lupu, 020021 Bucharest, Romania; ^3^“Carol Davila” Central Military Emergency Hospital, 134 Calea Plevnei, 010825 Bucharest, Romania; ^4^“Wolfson Medical Center”, 61 Halochamim Street, 58100 Holon, Israel; ^5^“Cantacuzino” National Medico-Military Institute for Research and Development, 103 Splaiul Independentei, 050096 Bucharest, Romania; ^6^“Prof. N. Paulescu” National Institute of Diabetes, Nutrition and Metabolic Diseases, 22-24 Gr. Manolescu, Bucharest 011233, Romania

## Abstract

Oral squamous cell carcinoma (OSCC) is the most common tumour of the oral cavity, associated with significant morbidity and mortality. It is a multifactorial condition, both genetic and environmental factors being involved in its development and progression. Its pathogenesis is not fully elucidated, but a pivotal role has been attributed to inflammation, strong evidence supporting the association between chronic inflammation and carcinogenesis. Moreover, an increasing number of studies have investigated the role of different mediators of inflammation in the early detection of OSCC. In this review, we have summarized the main markers of inflammation that could be useful in diagnosis and shed some light in OSCC pathogenesis.

## 1. Introduction

Oral cancer accounts for about 4% of all cancers. Histologically, over 90% of cases are diagnosed as oral squamous cell carcinoma (OSCC) [1]. OSCC is a destructive malignant tumour with an invasive behaviour and significant risk of metastases [2]. The most common localization is the tongue, but other frequent sites for tumour formation are the lips and floor of the mouth [3, 4]. OSCC is particularly diagnosed in the elderly, and although its pathogenesis is not fully understood, the main risk factors postulated include smoking, alcohol use, exposure to radiation and chemical carcinogens, infections, and immunosuppression [5–7]. With respect to infectious agents, a recent meta-analysis has shown that the human papillomavirus (HPV) genome is present in approximately one third of OSCC samples, HPV types 16 and 18 being the most commonly detected [8]. The most frequent premalignant lesions which can progress to OSCC are oral leukoplakia (OLK), oral lichen planus (OLP), and erythroplasia [5, 9]. A surgical approach of the tumour is the mainstay of treatment, but new therapies such as photodynamic therapy are promising, particularly in early-stage OSCC [10, 11].

The process of malignant transformation is complex and incompletely elucidated [12, 13]. The role of inflammation in carcinogenesis was first suggested by Rudolf Virchow in 1963 [14]. Since then, various studies have shown that chronic inflammation is a pathological response that can act to the detriment of the host and influences cell homeostasis and various metabolic processes, inducing changes even at the genomic level, which can promote carcinogenesis [15]. Moreover, several studies have suggested a pivotal role of chronic inflammation in carcinogenesis through the modulation of proinflammatory cells and cytokine production [12, 16]. In this review, we summarize the main mediators of inflammation that might be involved in the pathogenesis of OSCC and could represent biomarkers for the early diagnosis of the tumour.

## 2. Tumour Inflammatory Cell Infiltrate

The cells of the inflammatory infiltrate along with the mediators they release play an essential role in the formation of a suitable microenvironment that allows an uncontrolled cell proliferation [17, 18]. Two pathways of inflammation involved in the promotion of carcinogenesis, namely, the intrinsic pathway mediated by tumour cells and the extrinsic pathway mediated by tumour-infiltrating immune cells, have been described [19]. The tumour environment is an important entity, including both immature and adaptive immune cells; among them, the main role is played by tumour-associated macrophages (TAMs) and T lymphocytes. TAMs correlate with tumour progression, their presence in the inflammatory infiltrate being an unfavourable prognostic factor and represent an important source of cytokines [20]. Macrophages have the ability to release numerous enzymes and mediators which interfere with angiogenesis, cell proliferation, and metastasis. For example matrix metalloproteinases lyse the extracellular matrix and subsequently promote metastasis, and growth factors (epidermal growth factor (EGF), vascular endothelial growth factor (VEGF), fibroblast growth factor (FGF), etc.) favour cell proliferation [19, 21].

T lymphocytes can exert both inhibitory and stimulating effects on carcinogenesis through the cytokines they release; interleukin- (IL-) 6, IL-17, and IL-23 have a proinflammatory effect, favouring tumour progression, IL-12 and interferon-gamma (IFN-*γ*) exhibit an antitumour effect and tumour necrosis factor alpha (TNF-*α*), transforming growth factor beta (TGF-*β*), and IL-6 exert direct action on cell survival [20]. The development of a proinflammatory environment will enhance the interaction between tumour cells and their stromal cells that will underlie the initiation of carcinogenesis [22, 23]. Kullage et al. analysed the inflammatory infiltrate in OSCC and observed that lymphocytes represent the most abundant cell population. However, in poorly differentiated OSCC, the infiltrate was scarce. Therefore, they noticed a correlation between the infiltrate size and cell differentiation. This can be the result of the immunosuppression generated by malignant cells [24]. Malignant cells promote the migration of immunosuppressive cells into the tumour microenvironment resulting in the inhibition of immune response. The main recruited cells are regulatory T cells [25]. Thus, an increased number of lymphocytes in the inflammatory infiltrate is associated with a better prognosis while a decreased number of lymphocytes is associated with a lower survival rate. Other unfavourable prognostic factors include smoking, poorly differentiated tumours, and tumour localization (tongue) [26].

In order to establish the role of the inflammatory infiltrate, Mashhadiabbas analysed 125 samples from patients diagnosed with dysplasia (mild, moderate or severe) or OSCC, and found a positive correlation between the intensity of inflammatory infiltrate and lesion severity. The most abundant inflammatory infiltrate was observed among OSCC patients [27]. Lo Muzio et al. also studied the inflammatory infiltrate that accompanies OSCC and noticed a dense inflammatory infiltrate in well- and moderately differentiated tumours. In contrast, in the case of poorly differentiated tumours, a small amount of inflammatory infiltrate was revealed [28].

The study by Pellicioli et al. found an increased level of CD8 lymphocytes in OSCC compared to dysplastic lesions. In addition, they found a lower number of mature dendritic cells and an increased number of immature dendritic cells in OSCC samples compared to oral epithelial dysplasia (OED), a fact that might be involved in carcinogenesis [29]. Furthermore, Fang et al. have shown that the increased expression of CD8 lymphocytes in inflammatory infiltrate in OSCC specimens may be seen as a favourable predictor of survival. However, they have noticed that CD57 expression is a better survival marker than CD8 and CD4 [30].

Numerous studies have established the role of mast cells in allergies and inflammation, but recent research has shown that mast cells are also involved in angiogenesis and carcinogenesis [31]. Their role in tumour development and progression is debatable given that in some cancers, mast cell infiltration was correlated with a good prognosis but in others with an unfavourable outcome [32]. The role of mast cells in carcinogenesis is supported by the release of numerous cytokines, chemokines, and angiogenic factors [33–35]. Histamine, heparin, and FGF are among the most important mediators involved in angiogenesis [36].

The study by Kabiraj et al. revealed a positive correlation between mast cell infiltration and microvessel density in OSCC samples, suggesting the possible role of mast cells in tumour angiogenesis [37]. In contrast, the study by Kalra et al. showed a decrease in mast cell infiltration and an increase in the number of blood vessels from well-differentiated to poorly differentiated OSCC and concluded that mast cells are not the main regulators of tumour angiogenesis [36]. However, the study by Jain et al. highlighted that the presence of a high amount of mast cells and eosinophils in the tumour microenvironment is associated with a good prognosis in OSCC [38].

## 3. Markers of Inflammation in OSCC

To date, there are no reliable biomarkers for the detection of malignant transformation in early stages. In this sense, a series of markers have been studied; among them, markers of inflammation have attracted attention given the role of inflammation in carcinogenesis [39].

### 3.1. Systemic Inflammatory Response

An increasing number of studies have focused on the role of the systemic inflammatory response (SIS) as a prognostic factor in cancer [40]. The pathogenesis of SIS is unknown. However, some hypotheses have been postulated. It has been suggested that SIS may be the effect of the tumour hypoxia or necrosis or the consequence of the local tissue injury [41]. The most used biomarkers that reflect a SIS are white blood cell subtypes and C-reactive protein (CRP).

#### 3.1.1. NLR, LMR, and PLR

One of the most studied markers is the neutrophil-to-lymphocyte ratio (NLR). In many studies, NLR has been shown to be an unfavourable prognostic indicator for various diseases including neoplasia. Moreover, an increased NLR correlates with chronic inflammation [42]. A systematic review by Guthrie et al. showed that NLR is increased in patients with advanced and aggressive cancers [40].

Charles et al. performed a study on 145 patients with head and neck squamous cell carcinoma (HNSCC) and concluded that NLR can be used as a prognostic factor and a value higher than 5 is associated with a shorter survival; therefore, these patients should receive additional therapies that might prevent this outcome. In addition, it seems that NLR could be used to identify those patients at risk of relapse [43]. In line with this, in patients with oral cancer, Tsai et al. identified leukocytosis, monocytosis, neutrophilia, and elevated values of NLR associated with advanced cancer and undifferentiated tumour [44]. Moreover, Perisanidis et al. analysed 97 patients with OSCC with local invasion who were preoperatively treated with chemotherapy and observed that NLR is an independent marker for an unfavourable prognosis [45].

Eltohami et al. proposed SIS as a predictive factor in OSCC. To assess SIS, they determined the albumin level and lymphocyte-to-monocyte ratio (LMR). Low values of albumin level and LMR have been associated with advanced stages of the tumour. The low level of albumin was explained by the protein loss that accompanied advanced tumours [46]. Park et al. suggested a prognostic score system, which included the determination of NLR, LMR, and platelet-to-lymphocyte ratio (PLR). To assess the effectiveness of the score, they performed a study on 69 patients with OSCC and determined the aforementioned parameters. Low LMR and increased NLR and PLR were associated with high-grade lesions. Decreased LMR and increased NLR correlated with tumour size and increased PLR with significant lymph node involvement. These biomarkers could be useful in the follow-up of patients with OSCC [47].

#### 3.1.2. CRP

CRP is an acute-phase protein belonging to the pentraxin family. CRP is synthesized in the hepatocytes, primarily under the stimulation of IL-1 and IL-6. Elevated serum levels occur in case of inflammation, infection, or injury [48]. It is worth pointing out that a tumour can trigger an inflammatory response, resulting in the release of proinflammatory cytokines such as IL-6 and IL-1*β*. Thus, an increasing number of studies have revealed elevated levels of CRP in cancer [49, 50]. However, the direct link between CRP and cancer is still a debatable topic.

Allin et al. conducted a study that included 10,408 individuals, selected from the general population, and measured the serum levels of CRP. The subjects were followed for up to 16 years, and 1624 of them developed a type of cancer. The study demonstrated that elevated levels of CRP in individuals without cancer represent a risk factor for the development of a type of cancer [51]. In line with this, the meta-analysis by Guo et al. has shown that elevated levels of CRP are associated with an increased risk of cancer. In fact, the relationship between cancer and inflammation is bidirectional. The tumour leads to an inflammatory response including increased serum levels of CRP on the one hand, and chronic inflammation can be involved in the development of a malignant process, on the other hand [52].

The study by Metgud and Bajaj assessed serum and salivary levels of CRP in 20 patients with premalignant lesions, 20 OSCC patients, and 20 healthy subjects. Serum and salivary levels of CRP were highest in OSCC patients, and the lowest values were recorded in healthy subjects. CRP levels showed a graded increase according to the severity of the tumour. They suggested that CRP could be regarded as a prognosis marker in OSCC [53]. Khandavilli et al. highlighted that the elevated level of preoperative CRP is an unfavourable prognostic indicator [54].

Moreover, Chen et al. proposed CRP as a potential marker of OSCC aggressiveness. They found elevated levels of CRP in patients with advanced disease and lymph nodes or bone involvement. In addition, there was an association with the prognosis of the disease, patients with elevated CRP levels having a lower survival rate [55]. The same idea is supported by the study of Tai et al. on 343 patients with OSCC, which has shown a positive correlation between high CRP levels (≥5.0 mg/L) and oral cancer and revealed that the CRP level correlated with local and lymph nodes invasion. Regarding OSCC localisation, the best correlations were obtained in the case of buccal mucosa involvement [56].

Some investigators studied CRP in conjunction with other parameters. The study by Blatt revealed that CRP, hemoglobin, and ferritin could be used as biomarkers in prognosis and disease progression [57]. In the study by Park, the increased CRP/albumin ratio was associated with disease extension and a low survival rate, proving to be an accessible and useful marker for prognosis [58]. In addition, another study identified that elevated levels of CRP; a high number of total leukocytes, monocytes, and neutrophils; and a low number of lymphocytes correlate with a low survival rate in OSCC patients [59].

### 3.2. NF-*κ*B

The nuclear factor kappa-beta (NF-*κ*B) belongs to the Rel/NF-*κ*B family of transcription factors [60]. The activation of NF-*κ*B, a key event in the inflammatory process, is identified in various tumours and linked to carcinogenesis. NF-*κ*B has been shown to be involved in angiogenesis, inhibition of apoptosis, and cell proliferation [61, 62]. The activation of NF-*κ*B is initiated by various stimuli such as carcinogens, viral proteins, oncogenes, or infectious stimuli. The overexpression of NF-*κ*B has been identified in many tumours, and its suppression has been associated with the inhibition of cell proliferation and promotion of apoptosis [63].

However, the role of NF-*κ*B in carcinogenesis is controversial. The study by Piva et al. revealed a positive correlation between the overexpression of NF-*κ*B and the amount of inflammatory infiltrate in oral dysplastic lesions [64]. The study conducted by Bancroft emphasized that the increased expression of IL-1*α* in HNSCC induces the activation of transcriptional factors, NF-*κ*B and AP-1, and IL-8 expression. Furthermore, they observed that the epidermal growth factor receptor (EGFR) promotes NF-*κ*B activation in a murine model of SCC. Studies have shown that the inhibition of NF-*κ*B and EGFR is associated with beneficial effects in HNSCC resulting in the prevention of cell proliferation and stimulation of a cytotoxic response [65]. In contrast, van Hogerlinden et al. found that the inhibition of Rel/NF-*κ*B signalling in epithelial cells leads to an imbalance in cell development and differentiation with increased apoptosis of keratinocytes and the de novo appearance of squamous cell carcinomas. Thus, in a particular context, NF-*κ*B inhibition may play a role in the development of a malignant tumour [66].

The study by Alam et al. analysed whether there is a correlation between B-cell lymphoma protein 2 (BCL-2) gene expression and AP-1 and NF-*κ*B transcription factors. They evaluated samples of normal mucosa, primary oral tumour (PT), and recurrent chemo- and radioresistant oral tumour (RCRT). Regarding NF-*κ*B, the best correlation was observed with BCL-2 protein in the PT group and in the case of AP-1 in the RCRT group, suggesting the role of the two transcription factors in tumour progression or treatment resistance [67]. Wei et al. demonstrated that activation of NF-*κ*B and hedgehog signalling pathways is associated with lower survival in those patients with esophageal SCC [68].

### 3.3. Cytokines

Cytokines are small proteins that in the past were called lymphokines or monokines depending on the cells that produced them. It is now known that any nucleated cell has the ability to secrete cytokines, but their main cell source is represented by helper T cells and macrophages [69, 70].

Cytokines have a key role in modulation of the immune response and are classified into two broad groups, proinflammatory (IL-1, IL-6, IL-8, TNF-*α*, and TGF-*β*) and anti-inflammatory (IL-2, IL-12, IL-4, IL-10, and IFN-*γ*) cytokines [71]. Among the cytokines that may be involved in oral cancer, interleukins seem to have a crucial function, the most studied being IL-4, IL-6, IL-8, and IL-10 [72].

#### 3.3.1. Proinflammatory Cytokines – IL-6 and IL-8

IL-6 is synthesized in acute inflammatory response contributing to host defence. It has been shown to be involved in processes such as inflammation, immune response control, hematopoiesis, and oncogenesis. However, under certain conditions, elevated levels of IL-6 may lead to disturbances of the immune response [71, 73–75]. IL-6 can induce the transition from acute to chronic inflammation by recruiting monocytes to the site of inflammation through monocyte chemoattractant protein-1 (MCP-1) secretion [76].

Another proinflammatory cytokine which attracted the attention of investigators is IL-8, considered the prototype molecule in the chemokine class. IL-8 plays also an important role in the acute inflammatory response and persists for a relatively long time at the site of inflammation [77]. Its release from macrophages and neutrophils is activated by NF-*κ*B. Moreover, its expression is modulated by other various stimuli, such as inflammation, hypoxia, or steroid hormones. IL-8 binds to CRCX-1 and CRCX-2 receptors which have been identified both on inflammatory cells from the tumour-associated infiltrate and tumour cells [78, 79].

Wang et al. analysed 86 samples of OSCC and noticed higher expression of IL-6 receptor (IL-6R) and IL-6 mRNA compared to samples from tumour-free mucosa. The study revealed a positive association between IL-6R expression, tumour size, and histopathological stage. Moreover, expression of IL-6 mRNA was correlated with advanced disease (lymph node involvement and distant metastases) [80]. Sato et al. suggested that the posttreatment level of IL-6 might be a marker for the early detection of a locoregional recurrence [81]. In addition, it seems that IL-6 expression is associated with resistance to chemotherapy [82].

Schiegnitz et al. showed higher serum levels of IL-6, IL-8, and soluble IL-2 receptor (sIL-2R) in patients with OSCC compared to those with oral premalignant lesions and healthy subjects. Regarding the differentiation of oral premalignant lesions by OSCC, the best results were based on the IL-6 level. Higher sensitivity and specificity were obtained by measuring the levels of both IL-6 and IL-8. [83]. Similarly, Punyani and Sathawane analysed the salivary level of IL-8 in patients with premalignant oral lesions and OSCC and in a control group. They found a statistically significantly higher salivary level of IL-8 in patients with OSCC than in those with premalignant lesions. However, no statistical significance was obtained when comparing the premalignant group with the control group. They suggested that the salivary level of IL-8 could be used as a biomarker for OSCC, but not for premalignant lesions [84]. The study by Rao et al. showed that IL-8 could be involved in tumourigenesis by activating NF-*κ*B and STAT signalling pathways and concluded that chronic inflammation plays a key role in malignant transformation, IL-8 being a modulator of inflammation that should not be neglected [85].

Other research has also investigated the possible role of salivary cytokines in OSCC detection. The study included 9 patients with OSCC and 9 healthy subjects; TNF-*α*, IL-1*α*, IL-6, and IL-8 were measured. However, only the salivary levels of IL-6 were statistically significantly higher in patients with OSCC compared to the control group [39].

The study by Lee et al. on 41 patients with OSCC and 24 patients without oral malignant lesions evaluated an extended group of biomarkers in both plasma and saliva. The salivary determinations of eotaxin, IFN-*γ*, macrophage inflammatory proteins- (MIP-) 1*β*, IL-1*β*, IL-6, IL-8, and TNF-*α* were significantly higher in OSCC compared to controls. Regarding plasma determinations, significant differences between groups were observed only for IFN-*γ*-inducible protein 10 (IP10). Elevated plasma levels of eotaxin, granulocyte colony-stimulating factor (GCSF), and IL-6 were detected in advanced stages, and they have been proposed as markers of advanced OSCC [86].

In contrast, Czerninski et al. investigated the serum levels of IL-1, IL-6, IL-8, IL-10, and soluble IL-2 receptor in patients with precancerous oral lesions, OSCC, and post-OSCC status and they did not find statistically significant differences between the studied groups. They concluded that the investigation of serum levels of these parameters has little utility in the early detection of OSCC [87].

#### 3.3.2. Anti-inflammatory Cytokines - IL-10 and IL- 4

IL-10 is one of the most important interleukins with an anti-inflammatory role, being produced by numerous activated cells (B and T lymphocytes, macrophages, mast cells, etc.). IL-10 inhibits the release of proinflammatory mediators from macrophages and the antigen presentation [88]. IL-4 also exhibits an inhibitory effect on inflammation and angiogenesis and is particularly secreted by activated memory T cells [89]. IL-10 and IL-4 suppress the local immune response and mediate the recruitment of regulatory T cells and TAM in the tumour microenvironment [12]. Moreover, it seems that IL-4 may inhibit the invasion in oral cancers by decreasing matrix-metalloproteinase- (MMP-) 9 expression [90].

The study by Arantes et al., which focused on the analysis of anti-inflammatory cytokines in OSCC patients, showed a higher expression of IL-10 and TGF-*β*2 in OSCC samples compared to normal mucosa [91]. The same result regarding IL-10 was obtained by Alhamarneh et al., who suggested that IL-10 could be an unfavourable prognostic factor. The serum level of IL-10 was statistically significantly higher in OSCC patients compared to the control group [92]. Another study evaluated the role of immunosuppressive cytokines (IL-4, IL-10, IL-13, and IL-1 receptor antagonist-IL-1RA) in the early detection of OSCC on 30 patients with OSCC and 33 healthy subjects. Salivary levels of IL-10 and IL-13 were significantly higher in OSCC patients compared to normal subjects. Therefore, the study concluded that IL-10 and IL-13 could be used as biomarkers in OSCC diagnosis. In addition, it was highlighted that IL-1RA levels were higher in those with undifferentiated tumour compared to those with differentiated tumour [93]. Another study associated the increased expression of IL-10 in tumour cells with a more aggressive tumour phenotype [94]. In contrast, the study by Hamzavi et al. on 30 patients with HNSCC and 24 normal controls found no statistically significant differences between the two groups with respect to the salivary and serum IL-10 level. However, the tissue analysis revealed that IL-10 expression was positive in 86% of patients with OSCC and was not identified in controls [95]. A recent study has suggested that IL-10 could be involved in the immune evasion of tumour cells. A percentage of 91% of OSCC samples exhibited an increased expression of IL-10 [96].

Tsai et al. observed that there was an association between IL-4 genes -590 C/T polymorphism and oral cancer, suggesting that it might represent a marker in oral cancer detection [97]. The study by Sun et al. which assessed the expression of several cytokines in OLP, OLK, and OSCC revealed increased expression of IL-4 in OSCC compared to OLK and OLP and raised the hypothesis that IL-4 has a low immunosuppressive role in the tumour microenvironment [12]. The study by Beppu et al. highlighted that IL-4 can suppress NF-*κ*B activation and increase the production of MMP-9 in TNF-*α*-stimulated cells. Regarding IL-10, they did not observe these effects [90].

### 3.4. Cyclooxygenases

It has been observed that the association between increased expression of cyclooxygenase- (COX-) 2 and chronic inflammation is involved in the initiation of a carcinogenic process. Cyclooxygenases are enzymes that convert arachidonic acid to prostaglandins. Two main isoforms, COX-1 and COX-2, have been described [98]. COX-1 is expressed by the majority of cells and participates in various physiological events [99]. In contrast, under normal conditions, COX-2 is not expressed in the cells of the body, but under the influence of certain stimuli such as inflammation, it will be expressed [100]. Thus, COX-2 plays an important role in inflammation, but in recent years its role in carcinogenesis has also been studied. It seems that COX-2 is directly or indirectly involved in cell proliferation, inhibition of apoptosis, and angiogenesis [99, 101]. The role of COX-2 in carcinogenesis is also suggested by studies which have revealed a lower risk of developing a malignant tumour in those patients receiving COX-2 inhibitors [102]. Patel et al. pointed out that COX-2 overexpression could be correlated with the risk of recurrence of OSCC [103].

Sinanoglu et al. proposed COX-2 and Ki67 as biomarkers for the malignant transformation of oral leukoplakia. They revealed a higher COX-2 expression in OSCC samples compared to oral intraepithelial leukoplakia and oral hyperkeratosis, with a positive correlation between COX-2 levels and the severity of the lesions being observed. Similar results were found for Ki67 [104]. The study by Seyedmajidi et al. revealed higher COX-2 levels in OSCC compared to dysplastic and normal mucosal lesions. COX-2 levels showed a graded increase according to the severity of dysplastic lesions, but no correlation has been obtained with the severity of OSCC lesions [105]. In contrast, the study by Shibata et al. analysed normal, dysplastic, and OSCC samples and observed higher COX-1 and COX-2 levels in dysplastic lesions compared to OSCC; the levels increased according to the severity of the lesions, a fact that suggests the role of COX-1 and COX-2 as markers of early carcinogenesis. In OSCC lesions, a negative correlation between COX-1 and COX-2 levels and tumour histological grade was observed [106].

### 3.5. Matrix Metalloproteinases

Matrix metalloproteinases (MMPs) are a family of zinc-dependent enzymes, which encompass over 20 members with proteolytic activity that can disintegrate almost any extracellular matrix component. MMPs take part to many physiological processes such as tissue remodelling or regeneration but also in pathological conditions such as inflammation and tumourigenesis, by abnormal activation [107–109]. MMPs are involved in cell migration and regulate the level of cytokines at the site of inflammation, where, in turn, activated inflammatory cells release MMPs. It is worth noting that under such conditions, MMPs act on non-matrix substrates. It seems that they can activate or inactivate cytokine functions and subsequently promote or inhibit the inflammatory response [110]. Studies increasingly link MMPs to cancer [111, 112]. The main mechanisms postulated which support the hypothesis that MMPs participate in carcinogenesis include regulation of tumour cell growth and angiogenesis, modulation of apoptosis, extracellular matrix degradation, which facilitates tumour invasion, and epithelial to mesenchymal transition [107, 113]. Several researchers have analysed the role of MMPs as markers in OSCC.

The study by Chang et al., which investigated markers of inflammation in patients with oral leukoplakia and OSCC, showed that the most reliable markers are MMP-2, MMP-9, CRP, TGF-*β*1, and E-selectin. MMP-9 was the marker that best correlated with the evolution from normal tissue to leukoplakia and neoplasm. In addition, the study showed that the risk of relapse is greatest in patients with CRP ≥2 mg/L and E − selectin ≥ 85 ng/mL at baseline. The panel of the five studied markers was associated with a sensitivity of 67.4% for premalignant lesions and 80% for malignant tumours and a specificity of 90%, for the identification of the patients at risk of malignant transformation [114]. Another recent study has suggested that MMP-10 could also be a marker of malignant transformation of normal mucosa into OSCC [115].

Makinen et al. evaluated the expression of MMP-7 and MMP-25 in 73 patients with oral tongue SCC in an early stage. A percentage of 90% of the tumours expressed MMP-7 and MMP-25. The increased expression of MMP-7 was identified in poorly differentiated tumours and correlated with a higher risk of ocular metastases and a higher degree of local invasion. In contrast, MMP-25 expression did not correlate with any prognostic factors [116]. The study by Lawal et al. highlighted the increased expression of MMP-2 in poorly differentiated OSCC and a lack of expression of MMP-8; MMP-8 was identified in well-differentiated tumours. They suggested that MMP-2 could be a marker of tumour aggressiveness, and MMP-2 inhibitors could be used in therapy [117].

One of the phenomena that occur during the local invasion and subsequent metastasis is the degradation of the extracellular matrix and basal membrane [115]. Based on this fact, Tanis et al. evaluated the potential role of MMPs in metastatic OSCC. They included 12 patients with metastatic OSCC and 12 healthy controls and emphasized that the expression of MMP-1, MMP-3, MMP-9, and MMP-10 was greatest in those with metastatic OSCC, demonstrating the role of MMPs as early markers of metastatic tumour [118].

### 3.6. Galectins

Galectins are animal lectins with affinity for beta-galactosides. Galectins contain carbohydrate-recognition domains, which are involved in carbohydrate binding. Carbohydrate binding activity plays an important role in the various functions of galectins [119]. Numerous studies have investigated the expression of galectins in cancer patients. To date, according to the meta-analysis of Thijssen, there are over 200 studies, and most of them (>70%) have analysed the expression of galectin- (gal-) 1 and gal-3. With regard to the type of cancer, over half of the studies included patients with cancers of digestive or reproductive system [120]. In neoplasms, galectins have a dual role, pro- or antitumoural, depending on the type of cancer [121]. It seems that galectins modulate various biological processes, being regulators of adaptive immunity, homeostasis, tissue regeneration, and angiogenesis [122]. Galectins participate in immune response through diverse mechanisms, including the promotion of inflammation, stimulation of T cells, and modulation of regulatory T cell activity [9].

Noda et al. showed that the evaluation of the immunohistochemical expression of gal-1 in samples taken from the oral cavity could discriminate between neoplastic processes and reactive changes, decreasing the rate of false-positive and -negative results [123]. In addition, it could be used as a prognostic factor [124]. Aggarwal et al. proposed serum levels of gal-1 and gal-3 as screening markers for those patients at high risk of developing OSCC [125]. Another study has suggested the role of gal-9 in differentiating OSCC from premalignant lesions. Muniz et al. assessed the expression of gal-1, gal-3, and gal-9 in 40 OSCC samples and 40 premalignant lesions (20 OLP and 20 OLK) and 13 normal tissue samples. The expression of gal-9 was significantly higher in OSCC samples compared to premalignant lesions and healthy tissue. With respect to gal-1 and gal-3, the results were variable [126].

Mesquita et al. assessed the immunohistochemical expression of gal-3 and gal-7 in correlation with tumour clinical stage and histopathological grade in 32 patients younger than 45 years with OSCC. Gal-3 expression was identified in 65.6% of cases and did not correlate with the two studied parameters. In contrast, gal-7 expression was observed in a higher percentage, 96.9%, and a statistically significant correlation with the histological grade was obtained [127]. Another study suggested that gal-7 could be used as a marker of resistance to chemo and/or radiotherapy [128].

### 3.7. Markers of Oxidative Stress

Under physiological conditions, reactive oxygen species (ROS) exert beneficial effects being involved in the fight against infectious agents and preservation of cell homeostasis. However, under pathological conditions, the most common being conditions associated with chronic inflammation, ROS levels are increased and exhibit deleterious effects on cell components (lipids, proteins, and nucleic acids) [129–131]. The main consequence of chronic inflammation is an imbalance between oxidants and antioxidants, with increased production of ROS and decreased antioxidant protection. It was demonstrated that ROS are involved in carcinogenesis exerting effects on cell proliferation and apoptosis. It seems that ROS are the main chemical effectors acting at tissue level [132–135].

Huo et al. found elevated levels of ROS associated with low levels of antioxidant enzymes, in both blood and tumour tissue of the patients with OSCC. They revealed high levels of malondialdehyde (MDA) and nitric oxide (NO) and low levels of superoxide dismutase (SOD) and catalase (CAT) compared to the control group, suggesting the role of the imbalance between oxidants and antioxidants in carcinogenesis [136]. The same idea is supported by the study of Kumar et al. on 100 patients with HNSCC and 90 healthy subjects. The increased levels of ROS were associated with reduction of salivary total antioxidant capacity (TAC) and glutathione (GSH) level [137]. The study by Subapriya et al. revealed differences between the oxidative stress markers in tumour tissue and blood. Tissue determinations showed low lipid peroxidation in association with increased GSH-dependent antioxidant capacity. In contrast, increased lipid peroxidation and low antioxidant levels were detected in blood. It was therefore suggested that increased GSH-dependent antioxidant capacity represents the basis of a selective development of cancer cells which is detrimental to the normal cells. The results obtained from blood are explained by the sequestration phenomenon of antioxidants in the tumour cells and the susceptibility of erythrocytes to lipid peroxidation induced by ROS [138].

Manoharan et al. have identified that the levels of thiobarbituric acid reactive substances (TBARS) and antioxidants correlate with tumour status; there was a progressive increase in TBARS levels and a decrease in antioxidants levels from stage II to stage IV [139].

## 4. Conclusion

OSCC, a tumour with a local invasive behaviour and an important metastatic capacity, would benefit from an early diagnosis, which in turn may contribute to a more favourable outcome. Starting from the hypothesis that inflammation plays an important role in carcinogenesis, various markers have been investigated in order to elucidate the involvement of different inflammation pathways in OSCC pathogenesis. Thus, either common markers such as serum CRP or white blood cell subtypes, as well as more sophisticated markers such as MMPs or COX-2, may provide new insights into the molecular mechanisms of tumour development and progression and could open new pathways in diagnosis, prognosis, and therapeutic approach for OSCC. The quest for the ideal biomarkers in oral cancer is currently ongoing, and further studies are needed in order to establish the most reliable candidate markers with the greatest impact on both scientific research and clinical practice.
